# A High-Quality Reference Genome Sequence and Genetic Transformation System of *Aralia elata*

**DOI:** 10.3389/fpls.2022.822942

**Published:** 2022-03-01

**Authors:** Wenxuan Liu, Wenhua Guo, Song Chen, Honghao Xu, Yue Zhao, Su Chen, Xiangling You

**Affiliations:** ^1^State Key Laboratory of Tree Genetics and Breeding, Northeast Forestry University, Harbin, China; ^2^Key Laboratory of Saline-Alkali Vegetation Ecology Restoration, Ministry of Education, Northeast Forestry University, Harbin, China

**Keywords:** *Aralia elata*, genome assembly, evolutionary analysis, terpenoid biosynthesis pathway, transgenic system

## Abstract

*Aralia elata* is a perennial woody plant of the genus *Aralia* in the family Araliaceae. It is rich in saponins and therefore has a wide range of pharmacological effects. Here, we report a high-quality reference genome of *A. elata*, with a genome size of 1.21 Gb and a contig N50 of 51.34 Mb, produced by PacBio HiFi sequencing technology. This is the first genome assembly for the genus *Aralia*. Through genome evolutionary analysis, we explored the phylogeny and whole genome duplication (WGD) events in the *A. elata* genome. The results indicated that a recent WGD event occurred in the *A. elata* genome. Estimation of the divergence times indicated that the WGD may be shared by Araliaceae. By analyzing the genome sequence of *A. elata* and combining the transcriptome data from three tissues, we discovered important genes related to triterpene saponins biosynthesis. Furthermore, based on the embryonic callus induction system of *A. elata* established in our laboratory, we set up the genetic transformation system of this plant. The genomic resources and genetic transformation system obtained in this study provide insights into *A. elata* and lays the foundation for further exploration of the *A. elata* regulatory mechanism.

## Introduction

*Aralia elata* (Miq.) Seem. (Araliaceae), also known as Chinese angelica-tree, is widely distributed in Northeast China (mainly Heilongjiang and Jilin province), Korea, Japan, Russia, the south of Far East, the south of Sakhalin, and Kuril Islands (Ahn, 1998; Reunov et al., 2007). It is one of the most desirable mountain wild vegetables in Asia benefitting from the rich nutrients in its young shoots (Cheng et al., 2021). Furthermore, as a Chinese traditional medicinal plant, *A. elata* plays roles on rheumatism, diabetes, hepatitis, neurasthenia, and stomach spasms (Zhang et al., 2018), especially the anti-tumor role (Duan et al., 2019). Those medicinal potentials were depended on the bioactive components of saponins. More than 100 kinds of saponins belonging to triterpene saponins have been reported in *A. elata* and they are mainly oleanane-type saponins (Cheng et al., 2021).

The triterpenoid biosynthesis is initiated from isopentenyl diphosphate (IPP) that is derived from the metabolism of cytosolic mevalonic acid (MVA) or the plastid methylerythritol phosphate (MEP; Sawai and Saito, 2011). This biosynthesis process is catalyzed by a series of key enzymes. The enzymes before triterpenoid structural skeleton formation include farnesyl diphosphate (FPP) synthase (FPS), squalene synthase (SS), and squalene epoxidase (SE). Oxidosqualene cyclases (OSCs) catalyze oxidosqualene to different triterpenoid backbones. *β-AeAS* has been identified to encode the OSC in *A. elata* (Wu, 2011). Subsequently, the key enzymes are cytochrome P450 monooxygenases (P450), which mediate oxidations. Uridine diphosphate-dependent glycosyl transferases (UDT) finally catalyze glycosylations to generate different triterpenoid saponins (Sawai and Saito, 2011). Recent studies revealed that subfamilies of CYP450, such as CYP71, CYP72, CYP88, CYP93, CYP716, and CYP749 are extensively involved in the oxidative stress response (Heitz et al., 2012) and the biosynthesis of triterpenes (Carelli et al., 2011; Fukushima et al., 2011; Han et al., 2011), sterols, indole alkaloids (Irmler et al., 2000; Collu et al., 2001; Nafisi et al., 2007), geraniol iridoid (Höfer et al., 2013), etc.

In *A. elata*, genes that potentially encode these key enzymes, including *AeFPS* (Wu, 2011), *AeSS* (Cheng, 2011), *AeSE* (Zhao, 2012), and *β-AeAS* (Wu, 2011) have been cloned and investigated by real-time qRT-PCR. In addition, 254 members of P450 and 122 UGT families were identified by the RNA-sequencing analysis (Cheng et al., 2020). But for the complex pathway of triterpenoid synthesis, the information of these key enzyme encoding genes is still limited due to the lack of a genome reference of this species.

With the rapid development of sequencing technology and reduction of sequencing cost, more and more plant genomes have been sequenced and published. The third-generation sequencing, especially the High Fidelity (HiFi) technology, has greatly reduced the cost and shortened the circle of genome sequencing. In this study, we sequenced, assembled, and annotated a high-quality genome of *A. elata* using HiFi data. This is the first genome of the genus *Aralia*. Using comparative genomics, we explored the evolutionary trajectory and whole genome duplication (WGD) events of *A. elata*. We also identified the key enzyme encoding genes involved in the triterpenoid biosynthesis pathway in the genome. The expressional patterns of these genes were preliminarily investigated. Genetic transformation is the most efficient way to further explore the functions of the annotated genes in *A. elata*. However, no transformation system has been established for this non-model plant species. We therefore established an *Agrobacterium tumefaciens* mediated genetic transformation system for *A. elata*, which laid a solid foundation for plant genetic engineering. The genomic resources of *A. elata* provided here will be valuable for biological and breeding research on *Aralia* species and will provide new tools for Araliaceae geneticists and breeders.

## Materials and Methods

### Plant Materials, DNA Extraction, and Library Construction

Fresh and healthy leaves of 2-month-old tissue culture plantlets of *A. elata* were harvested and immediately frozen in liquid nitrogen and preserved at −80°C. The samples were then sent to the company (Annoroad Gene Technology, China) for DNA extraction. The quality and quantity of the isolated DNA were assessed using a NanoDrop 2000&8000 spectrophotometer and a Qubit 2.0 Fluorometer, respectively. Illumina and PacBio libraries were constructed using the eligible DNA following the instruction for each technology, respectively.

### Genome Sequencing, Assembly, and Quality Assessment

We integrated Illumina HiSeq and PacBio HiFi sequencing data to achieve the complete genome sequence of *A. elata*. The Illumina library was sequenced on the Illumina HiSeq X Ten platform following standard Illumina protocols. After filtering out adapter sequences, low-quality reads, and duplicated reads, the clean reads were used to investigate the genomic features including genome size and heterozygosity by *k*-mer distribution analysis using GenomeScope (Vurture et al., 2017). Two libraries were constructed for PacBio HiFi sequencing. The subreads generated from the PacBio libraries were assembled into contigs using hifiasm with the default parameters (Cheng et al., 2008). The Illumina sequencing reads were aligned to the genome assembly using BWA (Li, 2013) to assess its completeness. Benchmarking Universal Single-Copy Orthologs (BUSCO) was also used to assess the quality of the final genome assembly (Simão et al., 2015).

### Genome Annotation

The *A. elata* genome was annotated by the integration of multiple strategies including *de novo*, homology-based, and transcriptome-based predictions. Repeat Masker and Repeat Modeler were used to identify the repetitive sequences in the genome based on repeat sequence database. Augustus was used for *de novo* prediction of protein coding genes based on the repeat masked genome. For similarity-based gene prediction, eight species including *Arabidopsis thaliana*, *Oryza sativa*, *Daucus carota*, *Populus trichocarpa*, *Apium graveolens*, *Vitis vinifera*, *Panax notoginseng*, and *Coriandrum sativum* were selected, and the protein sequences of these species were downloaded from Phytozome.^1^ Annotation of coding genes in the genome was subsequently performed using these homologous proteins. BLAST with identity ≥ 0.95 and coverage ≥ 0.90 as thresholds was used to identify genes with significant similarity in the *A. elata* genome. To carry out the RNA-Seq aided gene prediction, we downloaded the transcriptome data of *A. elata* from NCBI SRA database (BioProject: PRJNA555256). The clean reads were assembled into transcripts using Trinity (Haas et al., 2013), which were aligned against the genome assembly for gene structure prediction using Program to Assemble Spliced Alignments (PASA; Haas et al., 2008). The gene sets predicted by the various strategies were integrated into a non-redundant and more complete gene set by Evidence Modeler (EVM; Haas et al., 2008). BUSCO was used to evaluate the integrity and completeness of the predicted gene set.

### Analysis of Genomic Evolution and WGD Events

We used OrthoFinder to identify the orthologous groups in 12 species: five species from Apiales including *A. elata*, *Panax notoginseng*, *Daucus carota*, *Apium graveolens*, and *Coriandrum sativum*, two species from Asterales including *Lactuca sativa* and *Taraxacum mongolicum*, one species from Tubiflorae (*Capsicum annuum*), three other dicot species including *Arabidopsis thaliana*, *Carica papaya* and *Populus trichocarpa*, and one monocot *Oryza sativa*, which was used as the outgroup (Guo et al., 2021). MUSCLE was used for multiple sequence alignment for each single-copy orthologous group identified by OrthoFinder (Emms and Kelly, 2019). All the alignment blocks were then manually concatenated, and substitution model for each alignment block was estimated using ModelTest-NG (Darriba et al., 2020) program. The results were subsequently used to construct a phylogenetic tree using maximum-likelihood algorithm. Divergence times of these species in the phylogenetic tree were estimated with MCMCtree (v4.0) using the Bayesian Relaxed Molecular Clock (BRMC) approach (Yang, 2007). The parameters of MCMCtree were set as follows: burn-in = 2,000; sample-frequency = 10; and sample-number = 20,000. *Oryza sativa* was designated as an outgroup of the phylogenetic tree. The calibration times of each divergent nodes were obtained from the TimeTree website (Kumar et al., 2017). Gene family amplification and contraction was analyzed by CAFÉ using the phylogenetic tree and gene numbers in each orthogroup (De Bie et al., 2006).

### Identification and Tissue Specific Expression of Genes Involved in Triterpene Saponins Biosynthesis

BLASTP, with E-value of 1e−5 as a threshold, was used to identify candidate enzymes that catalyze triterpene saponins biosynthesis. The NCBI Conserved Domain Database (Marchler-Bauer et al., 2010) was used to scan conserved domains in these candidates. Only the protein sequences containing canonical domains were identified as authentic enzymes. IQ-TREE (Nguyen et al., 2015) was used to construct phylogenetic trees for these protein sequences. The expressional profiles of genes encoding these enzymes were investigated using RNA-seq data retrieved from public databases (PRJNA555256). The gene expression analysis was performed using the nf-core/rnaseq v3.2 (Ewels et al., 2020) pipeline in nextflow v21.04.1 (Di Tommaso et al., 2017). The sequencing reads were mapped to the reference genome using Spliced Transcripts Alignments to a Reference V2.7.6a (STAR) as an aligner. Gene expression levels were then determined by using RNA-Seq by Expectation-Maximization v1.3.1 (RSEM). Trimmed mean of M value (TMM; Robinson and Oshlack, 2010) method was used to normalize and measure the expression levels of these samples.

### Establishment of *Agrobacterium* Mediated Genetic Transformation for *Aralia elata*

The system of vegetable propagation of *A. elata* was built in our lab (Dai et al., 2011). The somatic embryogenic callus was induced from the roots of the somatic embryo plants in the induction medium (1/2 SH medium with 3.0 mg/L of IBA and 0.2 mg/L of KT) for 3 weeks. When the callus was transformed to the re-differentiation medium: 1/2 SH with 1.0 mg/L IBA and 0.2 mg/L KT, after 6 weeks, lots of somatic embryo or plants emerged.

The roots of the above somatic embryo plants were used for *Agrobacterium tumefaciens* mediated genetic transformation. After 3 days of pre-culture, the roots were infected by *A. tumefaciens* for 5, 10, and 15 min, respectively and then co-cultured in medium for 3 days. Next, they were transformed to the selection medium with 50 mg/L kanamycin and 200 mg/L timentin. Then after 8 weeks, the calli were checked by PCR using the primers 5′-CGC ACA ATC CCA CTA TCC TT-3′, and 5′-AAG ACC GGC AAC AGG ATT C-3′ to choose the callus line of gene transformation. The positive callus lines were transformed into above plant-medium.

## Results

### Genome Sequencing, Assembly, and Annotation

**Figure 1:**
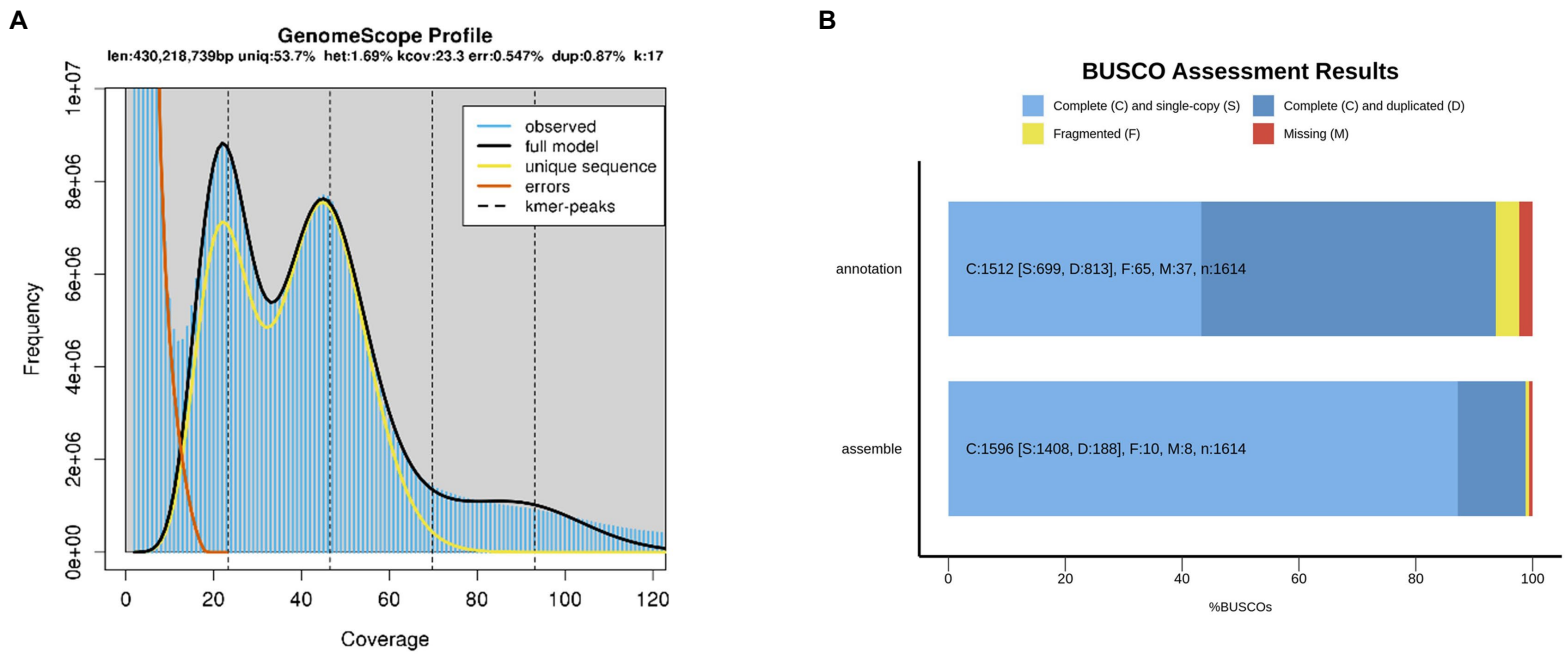
**(A)** 17-mer analysis for estimating the genome size of *Aralia elata*. **(B)** Assessment the gene coverage rate of genome assembly and annotation using Benchmarking Universal Single-Copy Orthologs (BUSCO).

To investigate the genomic features of *A. elata*, 17, 21, 25, and 27 *K*-mer distribution analysis was performed (Figure 1A; Supplementary Figure 1), respectively, using 56.89 Gb of the Illumina reads. The Illumina reads representing 50.79× coverage based on the estimated genome size of 1.12 Gb (*K*-mer analysis; Supplementary Table 1). The *K*-mer distributions followed a Poisson distribution, with two peaks corresponding to homozygous and heterozygous sequences, respectively (Supplementary Figure 1). According to the *K*-mer distribution analysis, the genome size of *A. elata* was estimated as 1.08–1.14 Gb and the heterozygosity ratio of the genome was estimated as 1.60–1.69% (Supplementary Table 2). The results indicated that the genome of *A. elata* is highly heterozygous and repetitive. We then used HiFi technologies to sequence the *A. elata* genome. A total of 51.14 Gb of HiFi reads from two libraries were obtained for the genome assembly. A total of 25.75 and 25.39 Gb data were generated from the two libraries, respectively (Supplementary Table 3). The HiFi reads were assembled into contigs using hifiasm. The final assembled genome was 1.21 Gb in size with a contig N50 length of 51.34 Mb. The genome assembly contained 1,350 contigs, the longest contig was 100.88 Mb, and the average contig length was 0.89 Mb. The GC content of the *A. elata* genome is 36.13% (Table 1).

**Table 1:** Assembly statistics of the *Aralia elata* genome.

N50 contig size (bp)	51,342,398
L50 contig number	8
N75 contig size (bp)	38,678,071
L75 contig number	15
N90 contig size (bp)	17,066,756
L90 contig number	22
Longest contig (bp)	100,882,612
Shortest contig (bp)	10,694
Average contig (bp)	893,717
Total length (bp)	1,206,518,707
Total N length (bp)	0
Number of contigs	1,350
GC content (%)	36.13

To evaluate the completeness of the genome assembly, short reads generated for the genomic survey were mapped to the genome. In total, 99.95% of the short reads were mapped to the contigs, 99.48% of which were properly pair-end mapped (Supplementary Table 4). The completeness of the genome assembly was also evaluated by BUSCO. The result revealed that the genome covered at least 98.8% of the BUSCO genes, 87.2% of which were classified as “complete and single-copy,” 11.6% as “complete and duplicated,” 0.6% as “fragmented,” and 0.6% as “missing” (Figure 1B; Supplementary Table 5). All the results suggested a high quality of the *A. elata* genome assembly.

Repetitive sequences, including tandem repeat and interspersed repeats, are important parts of genomes. In this study, two strategies, *de novo* prediction and homology-based identification, were used to annotate the repetitive sequences in the *A. elata* genome. According to the integrated results obtained above, the proportion of repetitive sequences in the genome was 71.69%, which was higher than carrot (45.95%; Iorizzo et al., 2016). The most abundant type of repetitive elements was long terminal repeat (LTR), which accounted for 49.15% of the genome, while DNA transposon repetitive sequences accounted for only 3.86% of the genome (Supplementary Table 6).

To annotate the protein coding genes in the *A. elata* genome, we used a combination of *ab initio* prediction, homology-based search, and transcript evidence from RNA-seq data. Finally, a total of 37,016 genes were annotated in the genome. We evaluated the completeness and quality of the annotated proteome through BUSCO using Embryophyta_odb10 as database. The results indicated that 97.7% of the conserved genes were annotated in the genome, which included 93.7 and 4.0% complete and fragmented BUSCO genes, respectively. The BUSCO assessment indicated that the annotation of genome was of high accuracy (Figure 1B; Supplementary Table 7).

### Genome Evolution of *Aralia elata*

**Figure 2:**
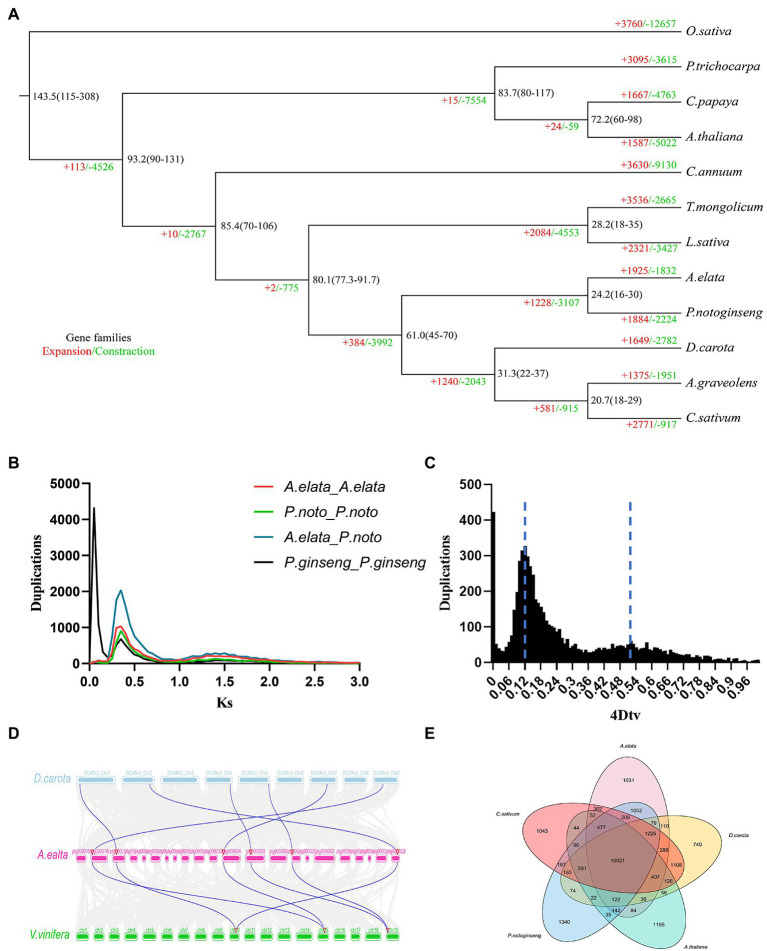
**(A)** Inferred phylogenetic tree of *Aralia elata* and 11 plant species based on protein sequences of single-copy orthologous genes. Numbers at each node represent the estimated divergence time of each node in million years ago (mya). Gene family expansions are indicated in red, and gene family contractions are indicated in green. **(B)** Ks distribution of paralogous gene pairs in the *A. elata*, *Panax notoginseng*, and *Panax ginseng* genome. The probability density of Ks was estimated using the “density” function in the R language. **(C)** Distribution diagram of 4DTv values. The dark black-filled part indicates the 4DTv analysis inside *A. elata*, and the peaks marked by the dotted line indicate where the two whole genome duplication (WGD) events of *A. elata* occurred. **(D)** Collinear analysis among *Daucus carota*, *A. elata*, and *Vitis vinifera* genome. The blue lines in the genomes of *A. elata* and *V. vinifera* indicate that the 2:1 correspondence between the two collinear regions. **(E)** Venn diagram showing the cluster distribution of shared gene families among *A. elata*, *C. sativum*, *P. notoginseng*, *D. carota*, and *A. thaliana*.

In order to reveal the evolutionary position of *A. elata*, we compared the genome assembly with genomes from 11 other plants. A total of 250 single-copy gene families were identified among these species by OrthoFinder. These single-copy genes were used to construct a phylogenetic tree using a maximum likelihood method. Consistent with Angiosperm Phylogeny Group, *A. elata* was closed to *P. notoginseng*, another Araliaceae species and these two species were classed into a clade. This clade was most closely related to the species from Apiales family (Figure 2A). The divergent times of these species were then estimated based on the phylogenetic tree. We estimated that *A. elata* and *P. notoginseng* diverged from Apiaceae at approximately 80.1 million years ago (mya). *Aralia elata* and *P. notoginseng* subsequently diverged into two species at around 24.2 mya. These results showed that the relationship between *A. elata* and *P. notoginseng* is very close. In addition, we performed a comparative analysis of gene family evolution in the phylogenetic tree. A total of 1,925 gene families were expanded in the *A. elata* lineage, whereas 1,832 gene families had undergone contraction (Figure 2A).

The gene family analysis among these species revealed that the 33,499 genes in the *A. elata* genome were clustered into 15,637 gene families with an average size of 2.14. The members in the gene families varied greatly, and the largest family contained 277 genes. We then investigated the specific and shared gene families among the species of *A. elata*, *C. sativum*, *P. notoginseng*, *A. thaliana*, and *D. carota*. The results indicated that 10,021 gene families were observed in all the investigated species, and 1,031 gene families appeared to be lineage specific to *A. elata* (Figure 2E).

Whole genome duplication occurs widely in flowering plants and plays important roles in genome evolution, the formation of new species, and gene neofunctionalization (Piegu et al., 2006; Van de Peer et al., 2009). The previous results indicated that two species in Araliaceae, *P. notoginseng* and *Panax ginseng*, have experienced one and two recent WGD events, respectively (Kim et al., 2018; Jiang et al., 2021). To further explore the evolutional trajectory of *A. elata*, we investigated the WGD events in its genome. The protein sequences from the *A. elata* genome were searched against themselves using BLASTP (E < 1e−5) to identify homologous gene pairs (Camacho et al., 2009). We calculated the 4DTv (4-fold degenerate synonymous sites of the third codons) for the optimal gene pairs and plotted the distribution of the 4DTv values (Figure 2C). Two peaks were observed at approximately 0.12 and 0.50, respectively. The right peak at approximately 0.50 revealed the core eudicot gamma triplication event. The left peak at approximately 0.12 indicated that *A. elata* underwent a recent WGD event. We then investigated the syntenic blocks between *V. vinifera* and *A. elata* using McscanX to further confirm the WGD event in *A. elata*, because *V. vinifera* does not undergo any recent WGDs. A 2:1 syntenic relationship between *A. elata* and *V. vinifera* (Figure 2D) was observed, which confirmed the recent WGD event occurred in the *A. elata* genome.

*Ks* (synonymous substitution rate) values can be used to estimate the timing of large-scale duplications (Blanc and Wolfe, 2004). We calculated the *Ks* values of the gene pairs and plotted the distributions to estimate the occurrence time of the WGD events of *A. elata*, *P. notoginseng*, and *P.ginseng*, respectively (Figure 2B; Chen et al., 2020). Two peaks were observed in the *Ks* distributions of the *P. notoginseng* and *A. elata* genomes, whereas the *P.ginseng* genome contained three *Ks* peaks. The *Ks* distribution result of *A. elata* was consistent with the 4DTv values. The main peak at approximately 0.38 indicated that a recent WGD event occurred in the *A. elata* genome. Similar *Ks* peaks around 0.38 were also found in the *P. notoginseng* and *P.ginseng* genomes, which indicated that the recent WGD event may be shared by *A. elata*, *P. notoginseng*, and *P.ginseng*. Then we calculated the occurring time of the WGD event of *A. elata* according to the method reported (Qin et al., 2014). The WGD event was estimated to occur at approximately 29.6 mya in the *A. elata* genome. Because the divergence time of *A. elata* and *P. notoginseng* was estimated to be 24.2 mya, this WGD event may occur before the differentiation of the two species. This is consistent with the published result (Jiang et al., 2021). All the results above indicated that unlike *P. ginseng*, who experienced an extra genus specific WGD, *A. elata* and *P. notoginseng* genome experienced only one recent WGD (Kim et al., 2018). In addition, this WGD may be shared by species in Araliaceae.

### Analysis of Key Enzyme Encoding Genes Involved in Triterpene Saponins Biosynthesis

**Figure 3:**
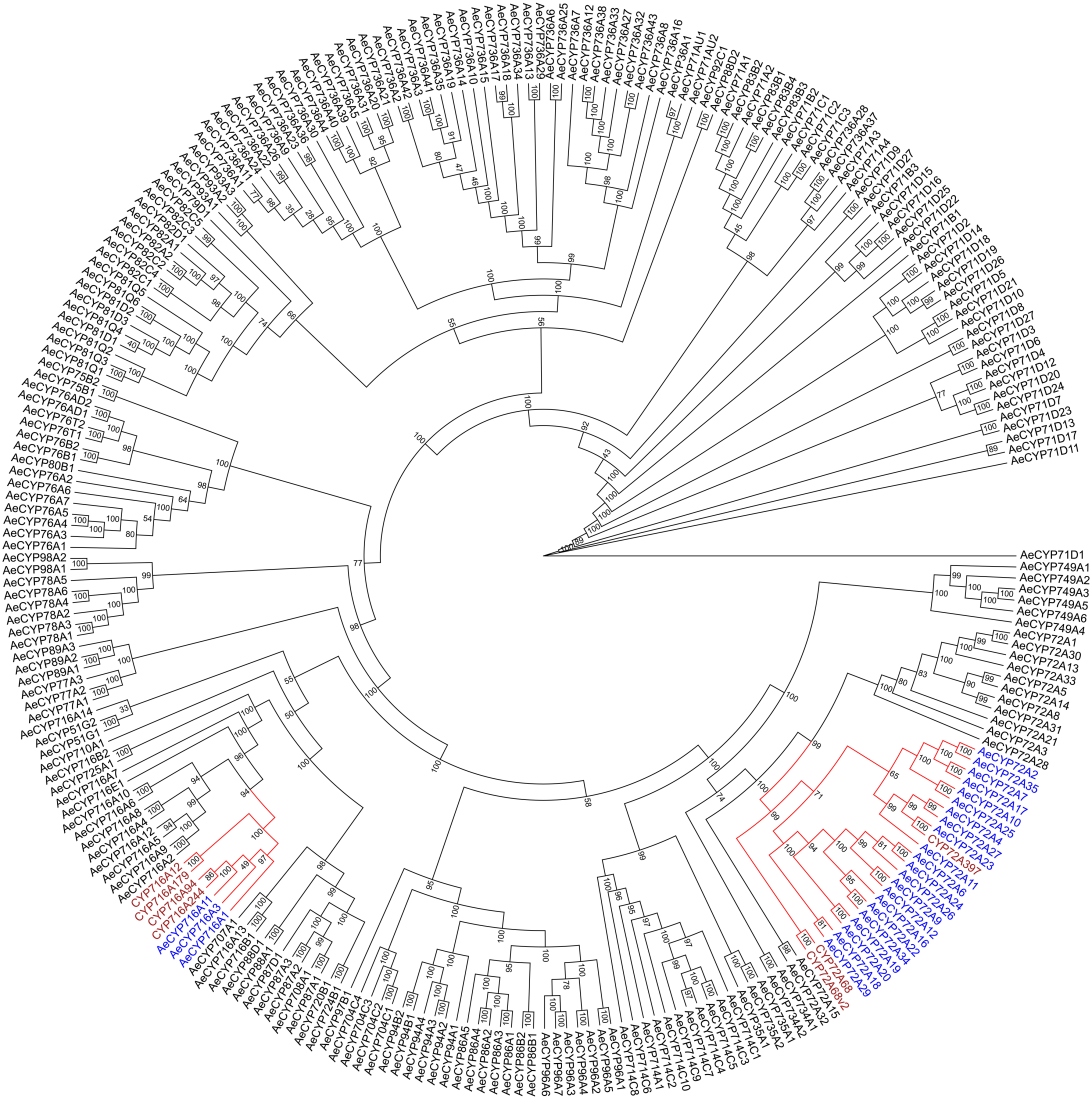
Phylogenetic analysis of P450 genes. Genes that putatively encode Cytochromes P450 were identified by the integration of sequence similarities, conserved domains, and the phylogeny. The colored clades in the phylogenetic tree include genes encoding the subfamilies of CYP72A and CYP716A, which are the key enzymes of P450 participated in the biosynthetic pathway of triterpene saponins. Genes marked in purple are reported to catalyze the synthesis of triterpene saponins in other species.

**Figure 4:**
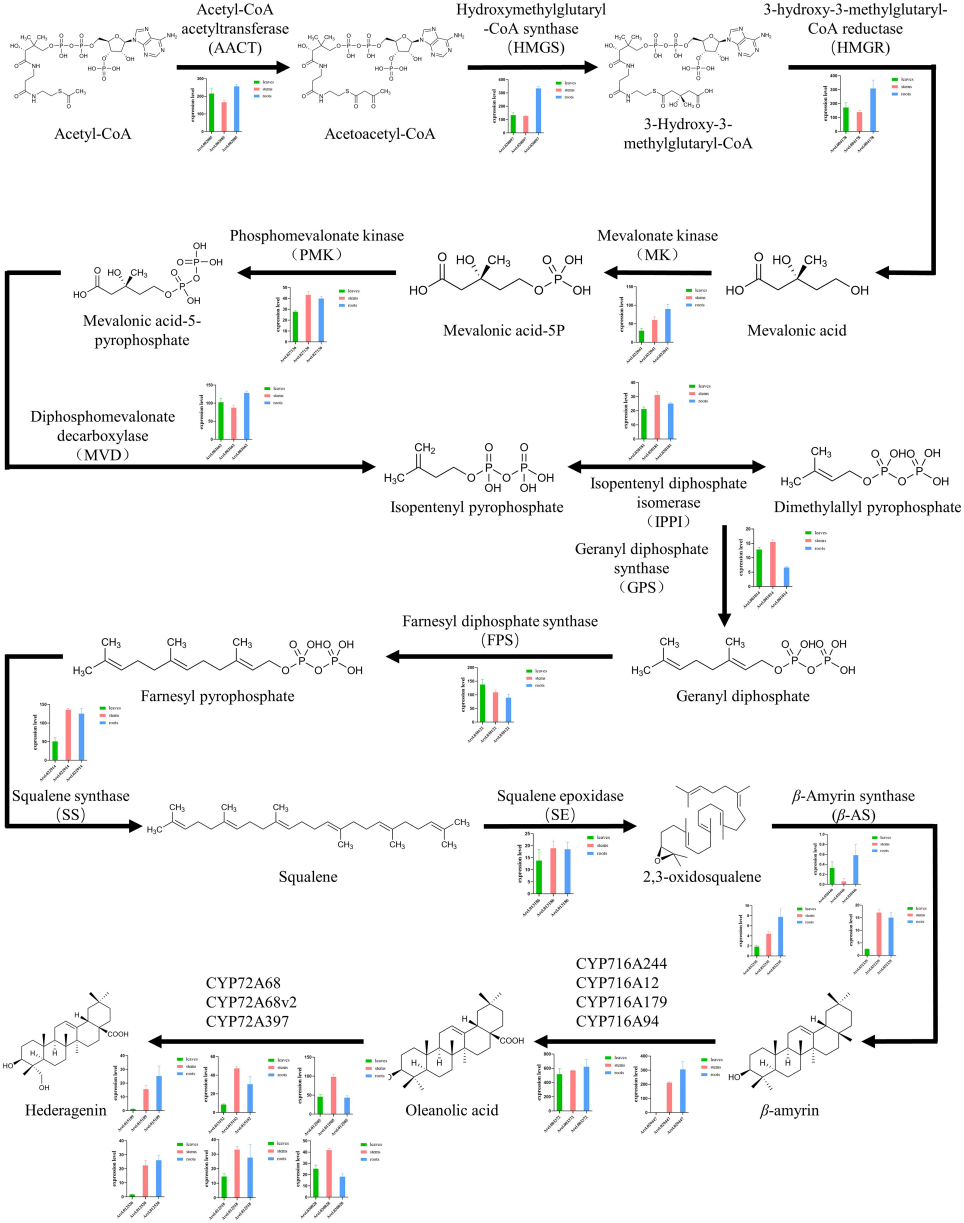
Overview of the saponin biosynthetic pathway in *Aralia elata* and expression profiles of key enzyme encoding genes. The histogram shows the expression levels of different genes in different tissues. Green indicates the amount of expression in the leaves, red indicates the amount of expression in the stem, and blue indicates the amount of expression in the root. The most genes of enzymes involved in the saponin biosynthesis pathway were highly expressed in roots higher than in leaves and stems.

The biosynthesis pathways of terpenoids in plants have been comprehensively explained. The research on *Aralia* Linn. plants have attracted extensive interest from researchers. By integrating sequence similarity, conserved domain, and phylogenetic relationship results (Figure 3; Supplementary Figure 2; Supplementary Table 8), we identified 22 candidate genes encoding the enzymes that may catalyze the biosynthesis processes of terpenoids in the *A. elata* genome (Figure 4). We used transcriptome sequencing data of *A. elata* downloaded from public database to investigate the expressional profiles of these genes. The RNA-seq reads were aligned to the genome assembly and obtained their expression levels in roots, stems, and leaves. Figure 4 illustrates the normalized expressional levels of these enzyme-coding genes in each tissue. The results indicated that many genes appeared to be expressed in tissue-specific manners. For example, genes encoding CYP450s are abundant in stems and roots.

Previous studies have shown that CYP72A and CYP716A subfamily members are the main CYP450s involved in the biosynthesis of pentacyclic triterpene saponins. Therefore, we pay special attention to the four CYP716a and three CYP72a coding genes identified in the *A. elata* genome. At the same time, 12 genes related to the terpene skeleton and triterpene biosynthesis pathway were identified, including MVA pathway and 2,3-oxysqualene biosynthesis pathway. Among them, six enzymes (AACT, HMGS, HMGR, MK, PMK, and MVD) were associated with the MVA pathway.

In the MVA pathway, Acetyl-CoA is synthesized into Acetoacetyl-CoA, which is catalyzed by the AACT enzyme (encoded by *Arel.002085*). The expression level of *Arel.002085* in leaves and roots are slightly higher than that in stems. Acetoacetyl-CoA is synthesized into 3-Hydroxy-3-methylglutaryl-CoA, which is catalyzed by HMGS enzyme (encoded by *Arel.020097*). HMGR enzyme (encoded by *Arel.004178*) subsequently catalyzes the synthesis of Mevalonic acid, and then MK enzyme (encoded by *Arel.022041*) is used to catalyze the synthesis of Mevalonic acid-5P. The expressional levels of *Arel.020097*, *Arel.004178*, and *Arel.022041* in roots were higher than those in stems and leaves, indicating that the biosynthetic reaction mainly occurred in the roots of *A. elata*. Then, under the catalysis of PMK enzyme (encoded by *Arel.027136*), Mevalonic acid-5-pyrophosphate was generated, and then Isopentenyl pyrophosphate is catalyzed by MVD enzyme (encoded by *Arel.003662*), and then catalyzed by IPPI enzyme (encoded by *Arel.030181*) to form Dimethylallyl pyrophosphate, further condensation of Isopentenyl pyrophosphate and Dimethylallyl pyrophosphate to form various terpenoids. Except for *Arel.003662*, whose expression levels in leaves and roots are slightly higher than those in stems, the expression levels of *Arel.027136* and *Arel.030181* in stems are slightly higher than those in leaves and roots. The results indicated that these biosynthetic reactions in this part may occur in the stems.

After that, Isopentenyl pyrophosphate and Dimethylallyl pyrophosphate is catalyzed by GPS enzyme (encoded by *Arel.001014*) to produce Geranyl diphosphate. FPS enzyme (encoded by *Arel.030122*) then catalyzes Geranyl diphosphate into Farnesyl pyrophosphate. SS enzyme (encoded by *Arel.022914*) catalyzes Farnesyl pyrophosphate into Squalene. Finally, 2,3-oxidosqualene is synthesized by the catalysis of SE enzyme (encoded by *Arel.013186*). In the synthetic pathway, *Arel.001014* and *Arel.030122* genes are expressed at higher levels in leaves and stems, while *Arel.002914* and *Arel.013186* genes are expressed at higher levels in stems and roots. Based on these results, it is speculated that the key triterpene skeleton biosynthesis reaction mainly occurs in the stem.

Finally, 2,3-oxidosqualene is catalyzed by *β*-AS (encoded by *Arel.032339*, *Arel.032335*, and *Arel.020446*) to form *β*-amyrin, and then oleanolic acid is formed under the catalysis of CYP716A subfamily members (including CYP716A244, CYP716A12, CYP716A179, and CYP716A94), and then Hederagenin is formed under the catalysis of CYP72A subfamily members (including CYP72A68, CYP72A68v2, and CYP72A397). Among them, genes encoding CYP72A and CYP716A subfamily members have higher expression levels in roots and stems than in leaves.

**Figure 5:**
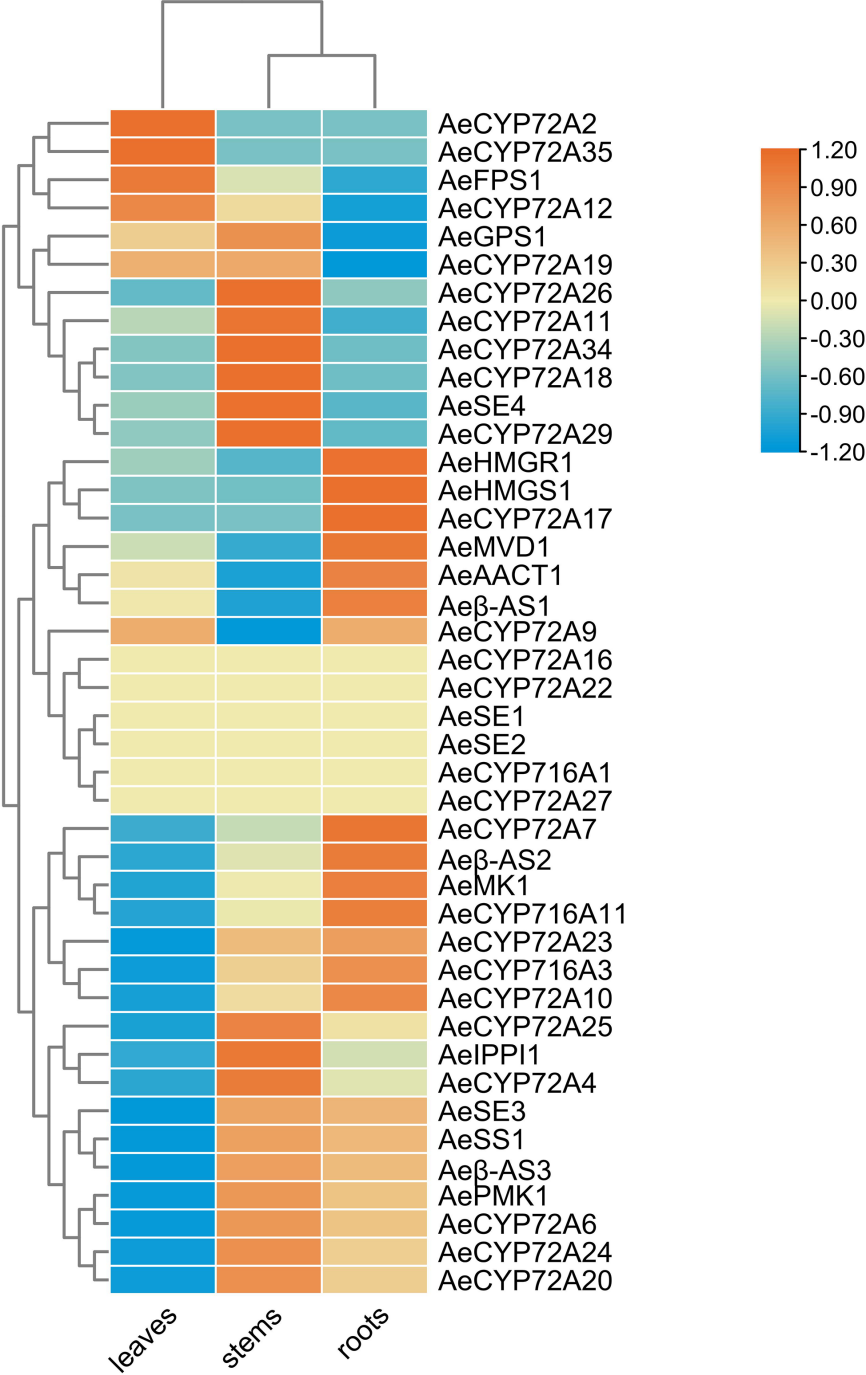
Heatmap of gene transcript abundance in the saponin biosynthetic pathway of key enzyme genes at three different parts in *Aralia elata*. RPKM values are log2-based. Yellow and blue indicate high and low expression levels, respectively.

Based on the expression levels of these genes, we explored the secondary metabolism in *A. elata* plants at the spatial levels. We compared the expressional patterns of these genes in different tissues. We found that most of the genes involved in the saponin biosynthesis were specifically expressed in roots, and a few were highly expressed in leaves and stems (Figure 5).

### Establishment of the *Agrobacterium* Mediated Transformation System for *Aralia elata*

**Figure 6:**
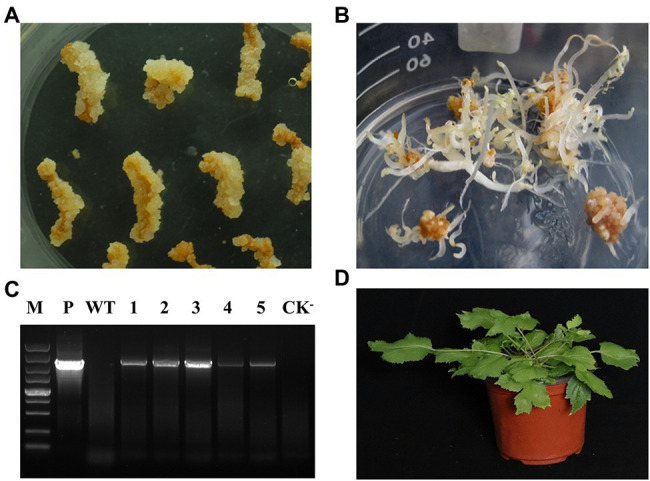
Establishment systems of the propagation and gene information of *Aralia elata*. **(A)** The induction of the somatic embryogenic callus from the root in the 1/2SH medium with 3.0 mg/L of IBA and 0.2 mg/L of KT for 3-week culture. **(B)** The somatic embryogenesis from that callus in the 1/2SH medium with 1.0 mg/L of IBA and 0.2 mg/L of KT for 3-week culture. **(C)** Introduction of the exogenous genes in the plant. P, positive control; The WT was not transformed with *Agrobacterium*; 1–5, the transgenic plant with target gene; CK^−^, negative control. **(D)** The somatic embryo plant of gene transformation.

Biotechnology is an efficient way to increase the contents of secondary metabolites in plants. The annotation of *A. elata* genome will provide many candidate genes for the generation of genetically modified *A. elata* plants. However, it is still difficult for genetic transformation in *A. elata*. Based on the embryonic callus induction system of *A. elata* established in our laboratory, we set up the genetic transformation system of this plant. Roots of well-grown tissue culture seedlings were used as explants for *A. tumefacien* infestation, and the roots were precultured, co-cultured, and selection cultured (kanamycin resistance) to obtain resistant callus. DNA extracted from the resistant callus was examined by PCR. As shown in Figure 6, the target fragment was successfully detected in the positive transgenic plants and found to be better transformed at 10 min of infection time. We transferred the transgenic callus to differentiation medium to obtain somatic embryonic seedlings. Next, the somatic embryo seedlings were transferred to WPM medium containing 20 g/L sucrose and cultured under 16 h light and 8 h dark conditions for 4 weeks, and then the plants were moved into soil and cultured in a greenhouse for 2 months, as shown in Figure 6, the transgenic plants grew well, and finally we obtained transgenic plants.

## Discussion

*Aralia elata* is one of the most widely used Chinese medicinal plants from the family Aralialeae and is well known in China and worldwide for its good efficacy. Triterpenoid saponins are widely existed in Aralialeae and are the most studied active ingredients of *A. elata*. Most of the aglycones are oleanolic acid, ivy, and their derivatives. Kochetkoy (1963) reported its chemical composition for the first time and obtained three saponins. The research on saponins of *A. elata* has become a hot topic, and many studies have reported its chemical components. Up to now, more than 100 saponins have been isolated and identified from *A. elata*. However, the complete biosynthetic pathway of saponins of *A. elata* has not been determined and further research is needed. Here, we briefly analyzed the terpenoid biosynthesis pathway of *A. elata*, to provide a reference for follow-up research. The contents of triterpenoid saponins in *A. elata* could be increased by genetically modification of the candidate genes involved in this pathway. The annotated genome and the genetic transformation system established in this study would be used for the further functional genome analysis in this species.

In the family Aralialeae, the genomes of some species including *Eleuthorcoccus senticusus* (Yang et al., 2021), *P. ginseng* (Kim et al., 2018), and *P. notoginseng* (Jiang et al., 2021) have been reported. The high-quality genomic analysis of *A. elata* will provide a valuable extensive information for studying the evolutionary landscape of other species in Araliaceae. Gene mining of high-quality genomic and transcriptomic data can provide resources for further exploration of plant growth and secondary metabolism mechanisms (Tu et al., 2020). So, we produced the first high-quality genome reference for *A. elata* with the latest sequencing technologies and bioinformatics methods. The size of the assembled genome is very close to the predicted result of *K*-mer, reflecting no obvious expansion or collapse occurred during the assembly process. Benefit from the long lengths and high accuracy of HiFi reads, the continuity and completeness of the *A. elata* genome obtained in this study are at a high-quality level. The evolutional process of the genome was studied based on the genome. Our results combined with the published genomes revealed the WGD trajectory in Araliaceae. A recent WGD event occurred before the divergence of species in Araliaceae.

In conclusion, the high-quality *A. elata* genome sequence described in this article, combined with comparative genome analysis, identification and tissue species expression analysis of putative genes involved in saponins biosynthesis, and the establishment of an efficient genetic transformation system of *A. elata* will contribute to *A. elata* breeding and cultivation.

## Data Availability Statement

The datasets presented in this study can be found in online repositories. The names of the repository/repositories and accession number(s) can be found in the article/**Supplementary Material**.

## Author Contributions

XY conceived the project. XY and SuC designed the experiments. WL, SoC, and WG performed most of the experiments and analyzed the data. The other authors assisted in the experiments and discussed the results. XY, SuC, WL, and WG wrote the manuscript. All authors contributed to the article and approved the submitted version.

## Funding

This work was financially supported by the National Natural Science Foundation of China (No. 30972390) and the Fundamental Research Funds for the Central Universities (2572018CL02).

## Conflict of Interest

The authors declare that the research was conducted in the absence of any commercial or financial relationships that could be construed as a potential conflict of interest.

## Publisher’s Note

All claims expressed in this article are solely those of the authors and do not necessarily represent those of their affiliated organizations, or those of the publisher, the editors and the reviewers. Any product that may be evaluated in this article, or claim that may be made by its manufacturer, is not guaranteed or endorsed by the publisher.

## Footnotes

^1^ https://phytozome-next.jgi.doe.gov/
