# Supplementary material for: A High-Quality Reference Genome Sequence and Genetic Transformation System of Aralia elata
Source: Front Plant Sci. 2022 Mar 1;13:822942. doi: 10.3389/fpls.2022.822942 (PMC8921765; doi:10.3389/fpls.2022.822942)

Supplementary Material

**Supplementary Tables**

**Supplementary Table 1.** NGS reads statistics of *Aralia elata*

|  | Total Reads | Data size(bp) | Q30（%） |
| --- | --- | --- | --- |
| Raw data | 380,001,336 | 57,000,200,400 | 88.12 |
| Clean data | 379,278,798 | 56,891,819,700 | 88.12 |

**Supplementary Table 2.** Estimation of genome size of *Aralia elata* based on *K*-mer analysis.

| k | *K*-mer total number | *K*-mer depth | Estimated genome size | heterozygosity | repeatability |
| --- | --- | --- | --- | --- | --- |
| 17 | 50,802,427,525 | 47 | 1,080,902,713 | 1.69% | 46.30% |
| 21 | 49,281,542,278 | 44 | 1,120,035,052 | 1.73% | 32.40% |
| 25 | 47,761,067,324 | 42 | 1,137,168,270 | 1.64% | 30.40% |
| 27 | 47,001,094,913 | 41 | 1,146,368,169 | 1.60% | 29.80% |

**Supplementary Table 3.** Sequencing data statistics of *Aralia elata*.

|  | HiFi-A | HiFi-B |
| --- | --- | --- |
| Total reads | 1,531,614 | 1,508,111 |
| Total base (bp) | 25,745,079,381 | 25,390,295,823 |
| Average length (bp) | 16,809 | 16,835 |
| Longest reads (bp) | 46,602 | 49,896 |
| L50 | 657,986 | 649,414 |
| N50 | 16,697 | 16,747 |
| L75 | 1,065,739 | 1,050,194 |
| N75 | 14,967 | 15,017 |
| L90 | 1,333,166 | 1,313,030 |
| N90 | 13,860 | 13,908 |
| GC (%) | 36.00 | 35.96 |
| Total reads (>= 100000 bp) | 0 | 0 |
| Total reads (>= 10000 bp) | 1,527,351 | 1,503,942 |
| Total reads (>= 1000 bp) | 1,531,605 | 1,508,103 |
| Total length (>= 100000 bp) | 0 | 0 |
| Total length (>= 10000 bp) | 25,714,105,002 | 25,361,056,754 |
| Total length (>= 1000 bp) | 25,745,073,957 | 25,390,294,053 |

**Supplementary Table 4.** Statistics of consistency assessment of the *Aralia elata* genome.

| Sample | *Aralia elata* |
| --- | --- |
| Raw Reads | 380,001,336 |
| Clean Reads | 379,278,798 |
| Clean Reads Rate (%) | 99.81 |
| Mapped Reads | 379,167,114 |
| Mapped Reads Rate (%) | 99.95 |
| Properly paired Reads | 377,316,826 |
| Properly paired Reads Rate (%) | 99.48 |

**Supplementary Table 5.** Assessment the gene coverage rate of genome assembly using BUSCO.

|  | Number | Percent (%) |
| --- | --- | --- |
| Complete BUSCOs (C) | 1,596 | 98.8 |
| Complete and single-copy BUSCOs (S) | 1,408 | 87.2 |
| Complete and duplicated BUSCOs (D) | 188 | 11.6 |
| Fragmented BUSCOs (F) | 10 | 0.6 |
| Missing BUSCOs (M) | 8 | 0.6 |
| Total BUSCO groups searched | 1614 | 100 |

**Supplementary Table 6.** Annotation of repetitive sequences in the *Aralia elata* genome.

| Type | Repeat length (bp) | % Of genome |
| --- | --- | --- |
| SINEs | 405,964 | 0.02 |
| LINEs | 15,647,329 | 0.77 |
| LTR elements | 998,312,339 | 49.15 |
| DNA transposons | 78,378,761 | 3.86 |
| Unclassified | 363,293,670 | 17.89 |
| Total | 1,456,038,063 | 71.69 |

**Supplementary Table 7.** Assessment the gene coverage rate of genome annotation using BUSCO.

| Iterms | Number | Percent (%) |
| --- | --- | --- |
| Complete BUSCOs (C) | 1,512 | 93.7 |
| Complete and single-copy BUSCOs (S) | 699 | 43.3 |
| Complete and duplicated BUSCOs (D) | 813 | 50.4 |
| Fragmented BUSCOs (F) | 65 | 4.0 |
| Missing BUSCOs (M) | 37 | 2.3 |
| Total BUSCO groups searched | 1614 | 100 |

**Supplementary Figure 1.** ***K*-mer analysis for estimating the genome size of *Aralia elata****.* (A) The 17-mer curve of *Aralia elata* genome (B) The 21-mer curve of *Aralia elata* genome (C) The 25-mer curve of *Aralia elata* genome (D) The 27-mer curve of *Aralia elata* genome.


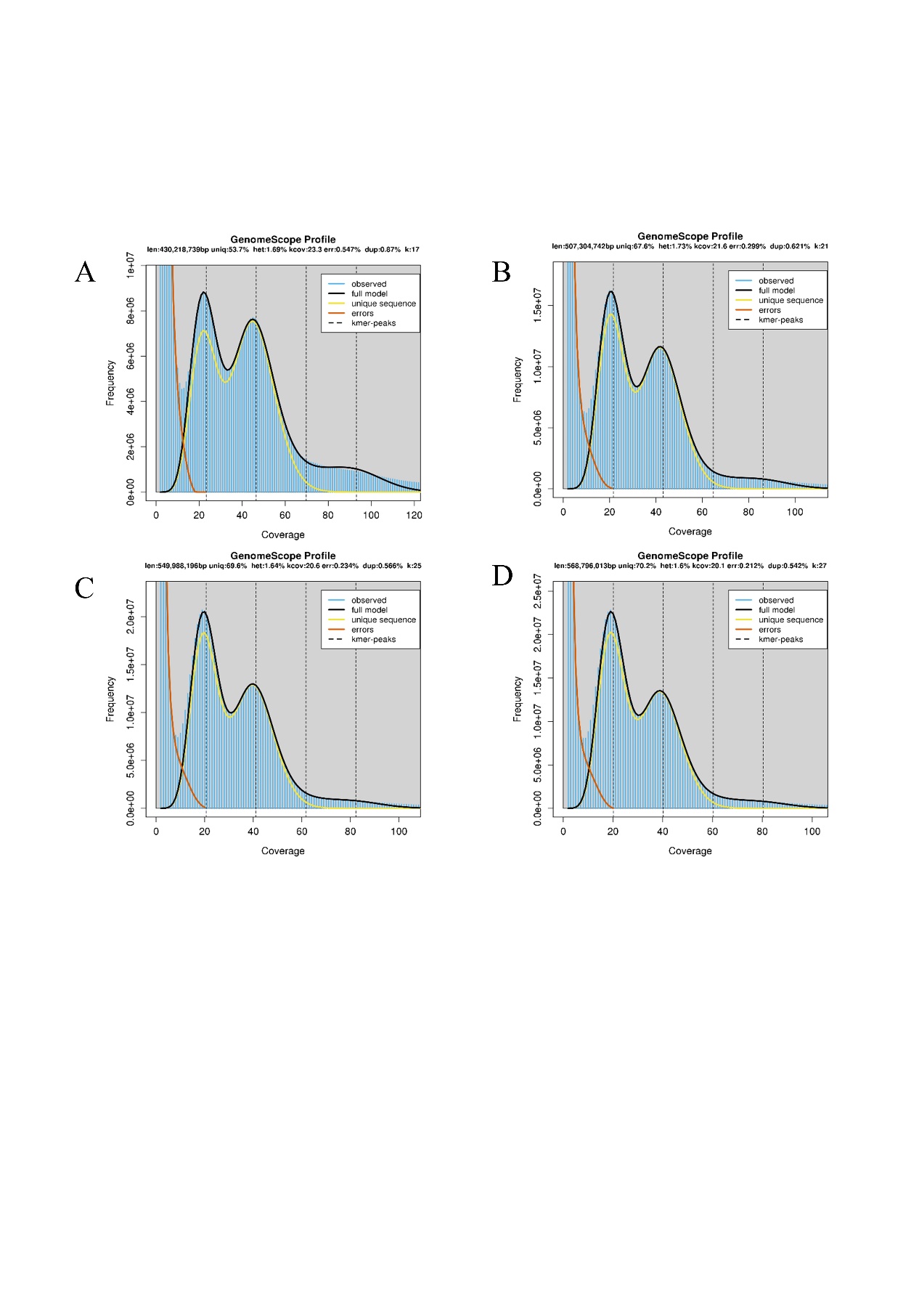


**Supplementary Figure 2. Identification and Phylogenetic analysis of genes in the saponin biosynthetic pathway in *A. elata*.**


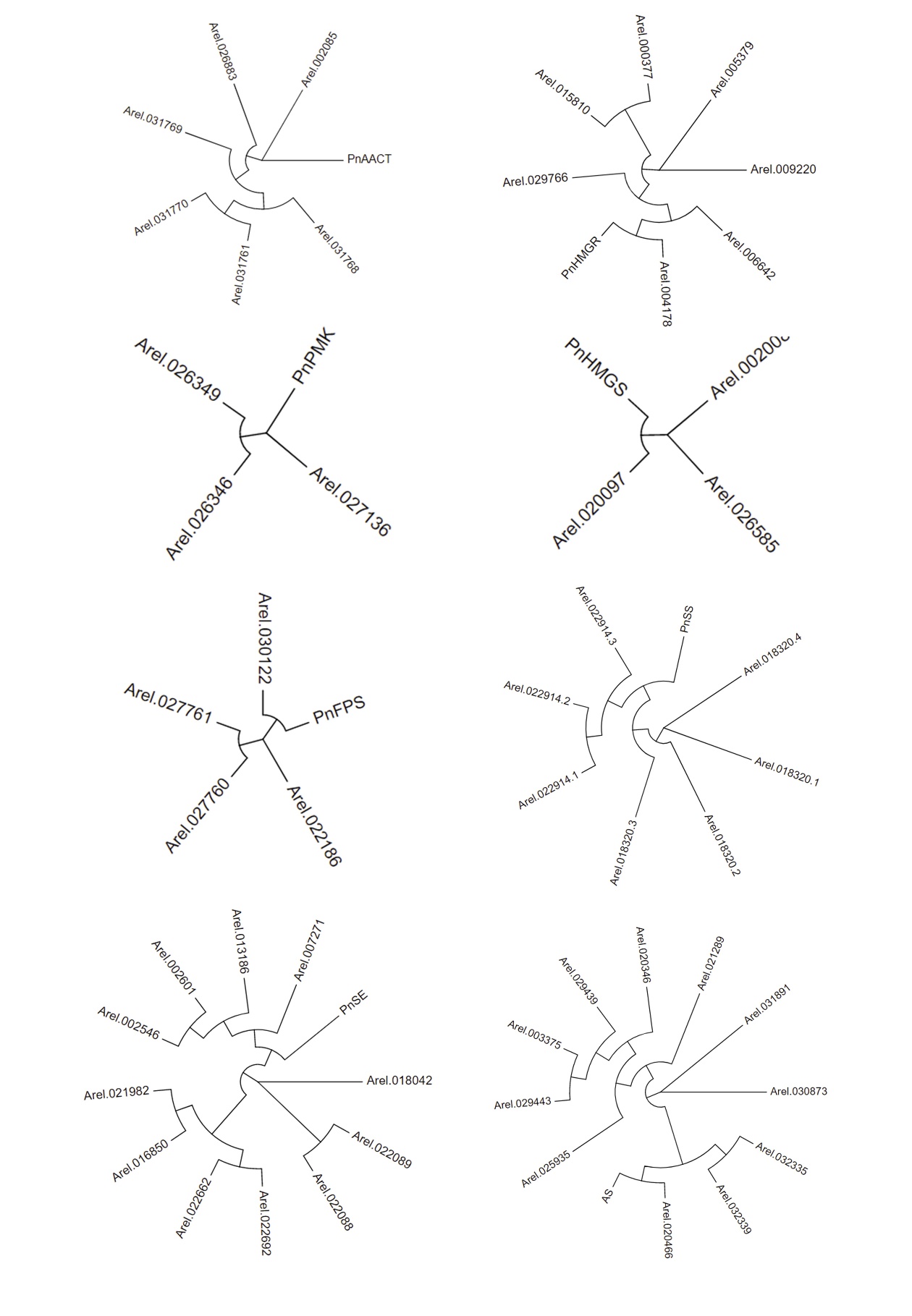

Supplement: Supplementary file 1 [file Table_1.DOCX]
